# Complex interaction and heterogeneity among cancer stem cells in head and neck squamous cell carcinoma revealed by single-cell sequencing

**DOI:** 10.3389/fimmu.2022.1050951

**Published:** 2022-11-14

**Authors:** Mintao Xiao, Xinyi Zhang, Duoli Zhang, Shuai Deng, Anfu Zheng, Fukuan Du, Jing Shen, Lin Yue, Tao Yi, Zhangang Xiao, Yueshui Zhao

**Affiliations:** ^1^ Laboratory of Molecular Pharmacology, Department of Pharmacology, School of Pharmacy, Southwest Medical University, Luzhou, China; ^2^ Cell Therapy and Cell Drugs of Luzhou Key Laboratory, Southwest Medical University, Luzhou, Sichuan, China; ^3^ School of Biomedical Sciences, Faculty of Medicine, The Chinese University of Hong Kong, Hong Kong, Hong Kong SAR, China; ^4^ South Sichuan Institute of Translational Medicine, Luzhou, Sichuan, China; ^5^ School of Nursing, Hunan University of Medicine, Huaihua, China; ^6^ School of Chinese Medicine, Hong Kong Baptist University, Hong Kong, Hong Kong SAR, China; ^7^ Department of Oncology, Affiliated Hospital of Southwest Medical University, Luzhou, Sichuan, China

**Keywords:** single-cell sequencing, head and neck squamous cell carcinoma, cancer stem cell, WNT signaling pathway, prognosis

## Abstract

**Background:**

Cancer stem cells (CSCs) have been characterized to be responsible for multidrug resistance, metastasis, recurrence, and immunosuppressive in head and neck squamous cell carcinoma (HNSCC). However, the diversity of CSCs remains to be investigated. In this study, we aimed to determine the heterogeneity of CSCs and its effect on the formation of tumor microenvironment (TME).

**Methods:**

We depicted the landscape of HNSCC transcriptome profile by single-cell RNA-sequencing analysis of 20 HNSCC tissues from public databases, to reveal the Cell components, trajectory changes, signaling network, malignancy status and functional enrichment of CSCs within tumors.

**Results:**

Immune checkpoint molecules CD276, LILRB2, CD47 were significantly upregulated in CSCs, enabling host antitumor response to be weakened or damaged. Notably, naive CSCs were divided to 2 different types of cells with different functions, exhibiting functional diversity. In addition, CSCs underwent self-renewal and tumor metastasis activity through WNT and ncWNT signaling. Among them, Regulon regulators (IRF1_394g, IRF7_160g, NFKB1_12g, NFKB2_33g and STAT1_356g) were activated in subgroups 2 and 3, suggesting their pivotal roles in the inflammatory response process in tumors. Among all CSCs, naive CSCs appear to be the most malignant resulting in a worse prognosis.

**Conclusions:**

Our study reveals the major signal transduction and biological function of CSCs during HNSCC progression, highlighting the heterogeneity of CSCs and their underlying mechanisms in the formation of an immunosuppressive TME. Therefore, our study about heterogeneity of CSCs in HNSCC can bring new insights for the treatment of HNSCC.

## Introduction

Squamous cell carcinoma of the head and neck (HNSCC) developed from the epithelium of the pharynx and oral mucosa is the sixth most common malignancy worldwide (1). The most closely associated causative factors for HNSCC include aging, exposure to environmental pollutants, family history, Epstein-Barr virus (EBV)/human papillomavirus (HPV) infection, and poor lifestyle habits, such as smoking and alcohol abuse (2). HPV (–) is the most common type of HNSCC and results in a worse prognostic outcome than (+) HNSCC (3). Oral cancer is the most common form of HNSCC and most often presents as oral squamous cell carcinoma. In addition to the above-mentioned causes, its causative factors also include vitamin A/E/C deficiency and dysbiosis of the oral microbiota (4). According to epidemiological studies and surveys, the occurrence of oral cancer in some Asia-Pacific populations is also associated with the chewing of betel nut (5). Currently, treatment for HNSCC includes surgical resection, radiotherapy, chemotherapy, molecular targeted therapy and immunotherapy (3). Immune checkpoint inhibitor (ICI) therapy is expected to be a promising treatment for HNSCC (6). Johnson et al. have reported the effect of pembrolizumab monotherapy in HNSCC is no less than the combination treatment of chemotherapy with cetuximab (3). However, the occurrence of drug resistance greatly hinders the continued benefit of immunotherapy for the treatment of HNSCC. In addition, several clinical studies have clearly demonstrated that the combination of immunotherapies targeting different immune checkpoints or the combination of immunotherapies with molecularly targeted drugs significantly increases the objective remission rate of patients. However, in the long term, the prognosis for HNSCC remains poor.

In the human body, stem cells are a kind of special cells that can differentiate into various cells and with the ability of self-renewal (7). However, the cancer stem cells (CSCs) are different from common stem cells in the human body. CSCs are more malignant, which means there is no limit to their proliferation. CSCs is based on the theory that tumor growth is similar to common tissues (8). Similarly, in cancer stem cells hypothesis, CSCs is a kind of cell supporting the growth of tumors. Some researchers even regard cancer as a disease that takes place in stem cells (9). CSCs promote tumor progression by contributing to tumor survival, proliferation, metastasis, recurrence and resistance to conventional treatment (10). For example, related studies have shown that CSCs upregulate the immune checkpoint molecule CD276 (B7-H3) to evade the host’s immune response (11). In addition, studies by Huang B. et al. have shown that targeting the Nanog and ERK1/2 signaling pathways can prevent or reverse the CSC phenotype and epithelial-mesenchymal transition that drive tumor progression, metastasis, and radiotherapy resistance in patients with HNSCC (12). Interestingly, the vast majority of studies have focused on CSCs as a whole and have not considered the intrinsic heterogeneity of CSCs. However, it is noteworthy that Ethan J. Kilmister et al. found, through molecular biology techniques, that different markers show different expression states in CSCs and confirmed the existence of three subpopulations (13). Collectively, scant research has been conducted to target the intrinsic heterogeneity of CSCs, and the specific functions and roles of subpopulations of CSCs are still unclear.

In this study, we used single-cell sequencing data to identify CSCs specific to HNSCC. Different subpopulations of CSCs were further explored by analyzing intercellular interactions and functional annotation of different cell populations. The cellular states of subpopulations of CSCs were redefined by analysis of their transcription factors and stemness. Finally, the ability of specific markers of CSCs to identify stemness features was validated in the HNSCC cohort of TCGA.

## Method

### Data and processing

The original RNA sequencing data and related clinical follow-up data were collected from The Cancer Genome Atlas (TCGA). Two groups of single-cell sequencing data from the GEO database, including 4 pre-treatment samples (GSE195832) and 16 samples (GSE103322), were used to construct the HNSCC tumor microenvironment (TME) atlas. In addition, a total of 300 HNSCC samples from the GSE65858 cohort were enrolled for survival analysis. 

### Determination of cell type, clustering, and annotation

Version 4.1.1 Seurat was used for downstream analysis. These samples from distinct oral cavities were integrated with CCA method to obtain a total of 33623 cells. Functions analysis of Quality Control, Normalization, Find Variable Genes, and PCA (first 20 principal components) were performed for further analysis. Both FindAllMarkers function and COSG (14) (COSine similarity-based marker Gene identification method) were used to accurately pick out the specific marker genes of each group. Then the marker genes were utilized to identify each group.

### Developmental trajectory inference

Version 2.22.0 monocle (15) was performed for developmental trajectory inference. Here, Monocle selected differentially expressed genes (DEGs) of the cell cluster to learn the sequence of gene expression changes each cell must undergo, thereby calculating the pseudotime. Subsequently, an individual cell was sorted according to the proposed pseudotime to simulate the dynamic cellular trajectory. In addition, we also applied a novel computational framework (CytoTRACE) (16) for predicting differentiation states (both in the cancer cell and cancer stem cell).

### Cell-cell interaction

Version 1.1.3 CellChat was used to investigate the cell-cell communication signal (17). We picked some pathways corresponding to our research, such as the MIF signaling network to show the cell-cell interactions of different groups.

### Enrichment analysis of gene sets

The gene sets of hallmarks involving six biological capabilities that occurred during the development of human tumors were included for enrichment analysis (18). There are nine kinds of methods to score the functional set. irGSEA was a rank-based integration framework for single-cell gene set enrichment analysis. In this research, we chose UCell to score each group in cancer cells to measure the expressions. Gene set variation analysis (version 1.42.0 GSVA) was performed using DEGs of each group in CSC by COSG function. Six gene sets associated with the CSC differentiation timescale were also enriched for KEGG signaling pathway and GO biological processes.

### Single-cell copy-number variation (CNV) evaluation

Two methods were applied to measure the CNV level: copykat (v1.0.8) (18) and inferCNV (v1.10.1) (19). Copy number karyotyping of aneuploid tumors was designed to distinguish non-malignant cell types from malignant cells *via* copykat. In inferCNV, cluster 4 (c4) was applied as the reference to determine if there is massive chromosome copy number variation in other cells in cancer cells.

### SCENIC for assessing the regulatory network analysis

SCENIC (Single-Cell Regulatory Network Inference and Clustering) was a computational method for gene regulatory network reconstruction and cell-state identification (20). The original motif datasets were downloaded from https://resources.aertslab.org/cistarget/ to construct co-expression networks and computationally infer the potential regulon of each cell. Scoring of each regulon activity of each cell using the AUCell algorithm.

### Statistical analysis

The whole statistical analysis process was achieved by R (v4.1.3) (http://www.r-project.org) language. Statistical difference between the two groups was evaluated using the Wilcoxon test. Kruskal-Wallis test was applied to assess difference among three or more groups. Kaplan-Meier survival curve was generated based on the median of stemness, and the log-rank test was utilized to evaluate the significance of differences. Statistical significance was defined as p < 0.05.

## Results

### Single-cell atlas and cell clustering of the TME in HNSCC

**Figure 1:**
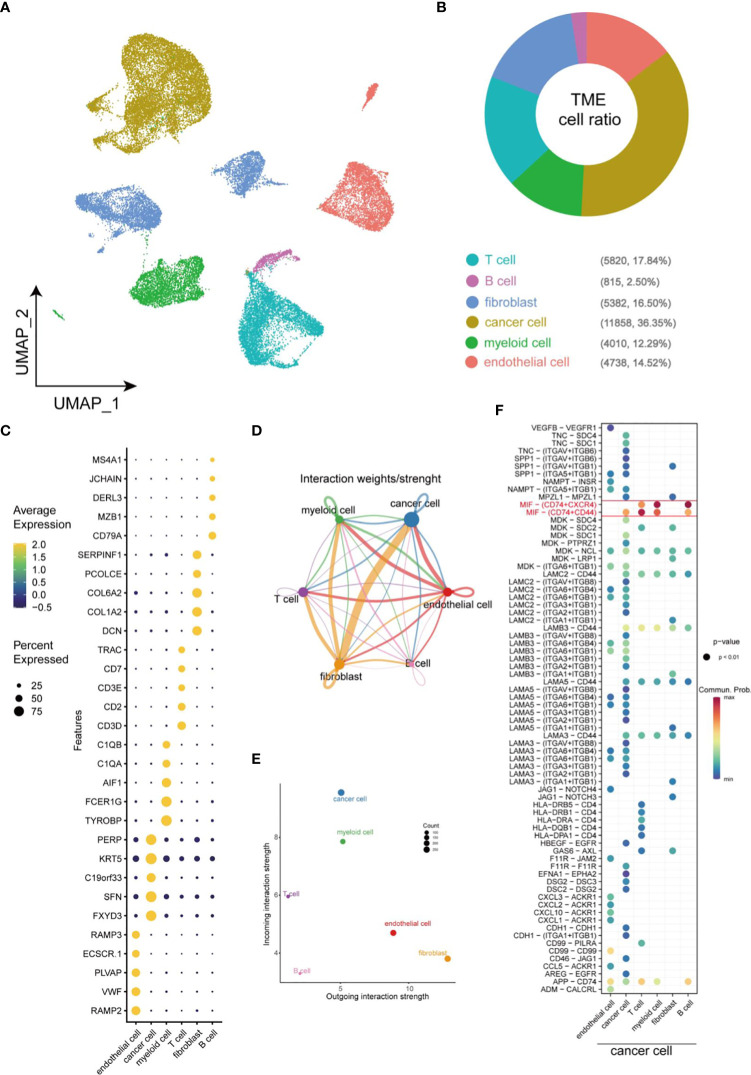
Overview of cellular heterogeneity of integrated single-cell expression profiling in HNSCC. **(A)** UMAP plot of 33623 cells in HNSCC. **(B)** Pie chart of the proportion of 6 types of cells in HNSCC. **(C)** Bubble plot of the top five highest expressed genes within 6 types of cells in HNSCC. The size of bubble represents the percentage of gene expression in the relevant cell types. **(D)** Weights/Strength of cell-cell interaction between different types of cells within HNSCC. **(E)** Output/input interaction strength of different types of cells within HNSCC. **(F)** Ligand-Receptor pairs between cancer cells and other cells in TME.

Single-cell sequencing was employed to generate single-cell profile to characterize the complexity within TME in HNSCC. A total of 33623 cells were isolated for further analysis after quality control. Uniform Manifold Approximation and Projection (UMAP), a nonlinear dimensionality reduction algorithm, was used to cluster these cells. Subsequently, specific genes expressed by each cell population were identified by COSG algorithm analysis. Finally, a total of 6 cell populations were identified according to the expression level of these marker genes (**Figure 1A**). Within these cells, non-immune cells are main components, including cancer cells (36.35%), endothelial cells (14.52%), and fibroblasts (16.50%), while immune cells (such as T cells (17.84%), myeloid cells (12.29%), B cell (2.50%)) only account for 32.63% of the total cells (**Figure 1B**). A dot plot was employed to visualize the results of the top 5 most significant gene expression levels in COSG (**Figure 1C**). **Figures 1D, E** depicted the overall cell-cell interactions of the six cell clusters within HNSCC. Notably, fibroblasts and cancer cells presented the strongest output and input signals, suggesting their essential role in TME. A comparison of the communication between cancer cells and other cells in TME elucidated that MIF-CD74/CXCR4 and MIF-CD74/CD44 were the strongest Ligand-Receptor (LR) pairs between cancer cells and immune cells (**Figure 1F**).

### Identification of CSCs in HNSCC

**Figure 2:**
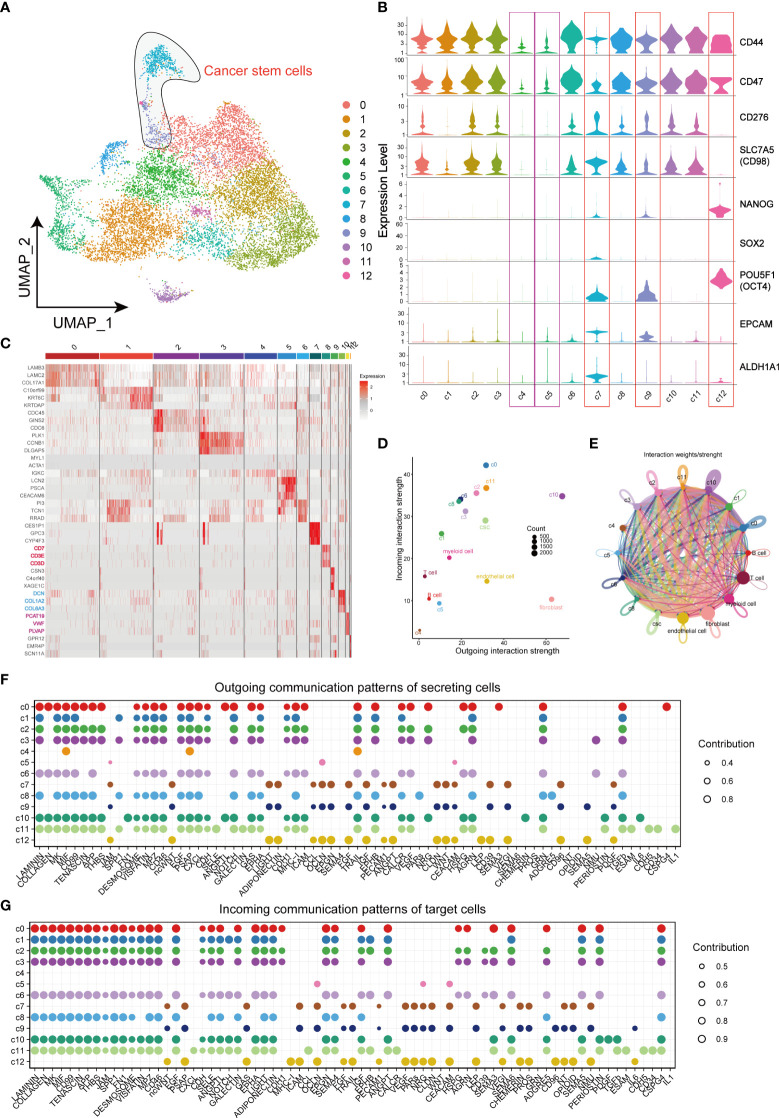
Landscape of characteristics of different clusters in cancer cells characterized by single-cell transcriptomic sequencing. **(A)** UMAP plot of 11858 cells in subgroup of cancer cells. **(B)** Violin plots of gene expression patterns of 13 cluster of cells types in subgroup of cancer cells. **(C)** Heatmap of the top three highest genes within 13 clusters of cancer cells. **(D)** Output/input interaction strength of different clusters of cancer cells and other types of cells in TME. **(E)** Weights/Strength of cell-cell interaction between different cell clusters of cancer cells. **(F)** Outgoing communication patterns of secreting cells. **(G)** Incoming communication patterns of secreting cells. Bubble size represents the strength of the signal.

For the purpose of identifying CSCs, data of cancer cells were extracted separately for further dimensional reduction clustering. Finally, a total of 13 clusters (from C0 to C12) was identified (**Figure 2A**). In previous studies, CD44, CD98, CD47, CD276, EPCAM, ALDH1A1 and transcription factors including NANOG, SOX2 and OCT4 were regarded as markers of CSCs. CD44, CD98, CD47, and CD276 were enriched in CSCs and promoted CSCs phenotypes maintenance (11, 21–23). High expression of EPCAM and ALDH1A1 in CSCs also enhances invasiveness leading to poor prognosis in HNSCC patients (24–26). Another 3 types of transcription factors constituted the core transcriptional network and were responsible for regulating CSC self-renewal and pluripotency (27). Violin plots were applied to visualize the mRNA level of these CSCs markers, and the results suggested CSCs-associated genes mainly enriched in cells of C7, C9 and C12, but not enriched in C4 and C5, which indicating the distribution of CSCs in TME (**Figure 2B**). Thereafter, the heatmap visualized the markers of each cluster based on its gene expressions (**Figure 2C**). Notably, cells of C8 subgroup were T cell-like cancer cells and expressed specific markers of T cells, such as CD7, CD3D, and CD3E. Similarly, fibroblast-like cancer cells (C10) and endothelial cell-like cancer cells (C11) were identified based on the specific expression of fibroblast markers (DCN, COL1A2 and COL6A3) and endothelial cell markers (PCAT19, VWF and PLVAP). CellChat analysis of interactions among all the cells in the TME showed little interaction between C4 and other cells, and the cells of C5 subgroup showed similar characteristics with C4 cells (**Figures 2D, E**). Notably, these 2 clusters did not express any stemness related genes and were defined as CD44^-^ cancer cells. In contrast, fibroblast-like cancer cells were with most intensive signal exchange in the TME, characterizing both extremely strong incoming signals of tumor cells and signals of outgoing fibroblasts (**Figures 2D, E**). In addition, we identified the communication patterns of signal output cells and signal input cells by NMF method (**Figures 2F, G**). CD44^-^cancer cells (C4, C5), CSCs (C7, C9, and C12) and other CD44^+^ cancer cells (C0, C1, C2, C3, C6, C8, C10, and C11) presented different signaling communication patterns in TME, demonstrating intra-tumor heterogeneity.

### Complex cell-cell interaction between the CSCs and other cells in the TME

**Figure 3:**
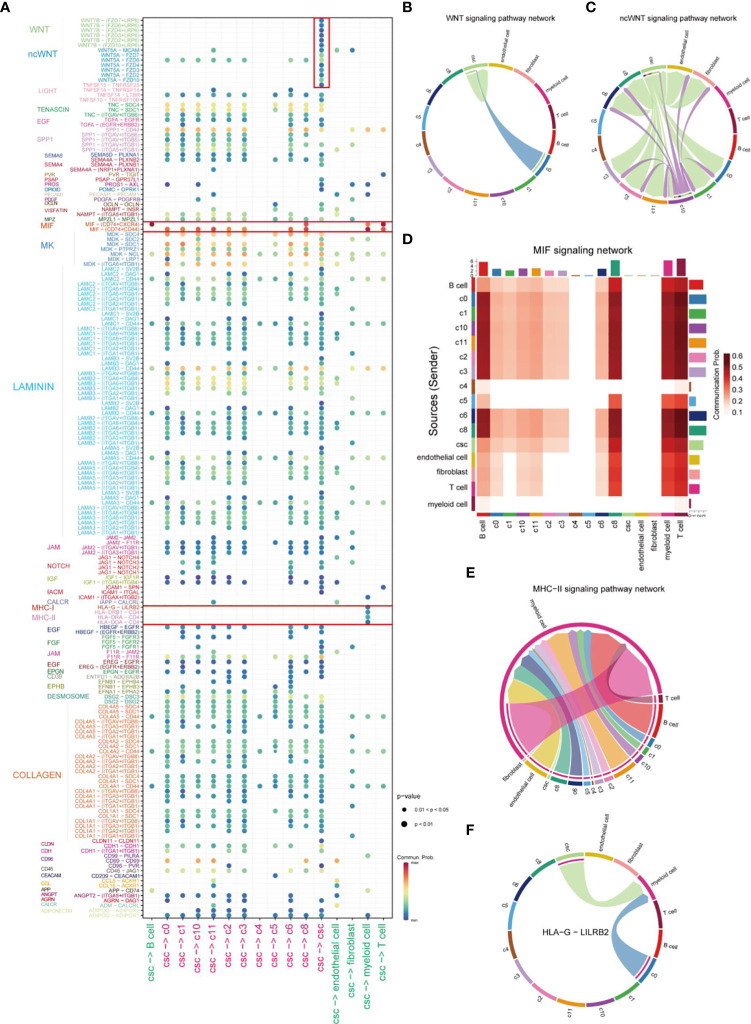
Comprehensive analysis of cell-cell interactions in HNSCC. **(A)** Ligand-receptor pair association analysis of cellular-cell interaction between CSC and other cells in HNSCC. **(B)** Plot of cells which interacted *via* WNT signaling pathways in HNSCC. **(C)** Plot of cells which interacted *via* ncWNT signaling pathways in HNSCC. **(D)** Cells involved in MIF signaling networks in HNSCC. **(E)** Plot of cells which interacted *via* MHC-II signaling networks in HNSCC. **(F)** Signal transduction of HLA-G-LILBR2 receptor-ligand pair of HLA signal in HNSCC.

After identifying the clusters of CSCs from cancer cells, we further investigated the mechanisms of interactions between CSCs and other cells in the TME. The bubble plot displayed the LR pairs of CSCs interaction with other cells in TME, indicating that the effect of CSC on tumor cells was much stronger than other stromal cells (**Figure 3A**). In the LR pairs between CSCs with other cells in cancer cells, LAMININ and COLLAGEN signaling networks showed strongest interactions. Canonical WNT signaling in CSCs worked in an autocrine way (**Figure 3B**), promoting the maintenance of stemness via affecting the proliferation and differentiation capacity of CSCs (28, 29). CSCs, in concert with C10, were involved in non-canonical WNT signaling cascades in CD44^+^ cancer cells, endothelial cells and fibroblasts (**Figure 3C**). Macrophage migration inhibitory factor (MIF) signaling network, as the strongest signal from CSCs, which was mutually interacted by different LR pairs, mediated the cell-cell interaction between tumor cells and immune cells. However, There was no significant difference in the capacity of emitting signals between CSCs and other CD44^+^ cancer cells (**Figure 3D**). An analogous phenomenon was observed in the major histocompatibility complex (MHC) II signaling network (**Figure 3E**). It was reported that MHC class I and class II molecules regulate NK cell and T cell functions via binding to receptors LILRB2 and CD4 (30, 31). In this study, our results also suggested CSCs affected myeloid cells through the above receptor ligands (**Figures 3E, F**). The interactions between Myeloid cells, endothelial cells, CD44 cells (except CSCs) and cells of C10 and C11 subgroup might associated with drug resistance in HNSCC (32) (**Supplementary Figure 1A**). In addition, CSCs, C10 cells and endothelial cells could work as effector cells to promote tumor progression via VEGF signaling pathway (**Supplementary Figure 1B**).

### Complex heterogeneity within tumor cells

**Figure 4:**
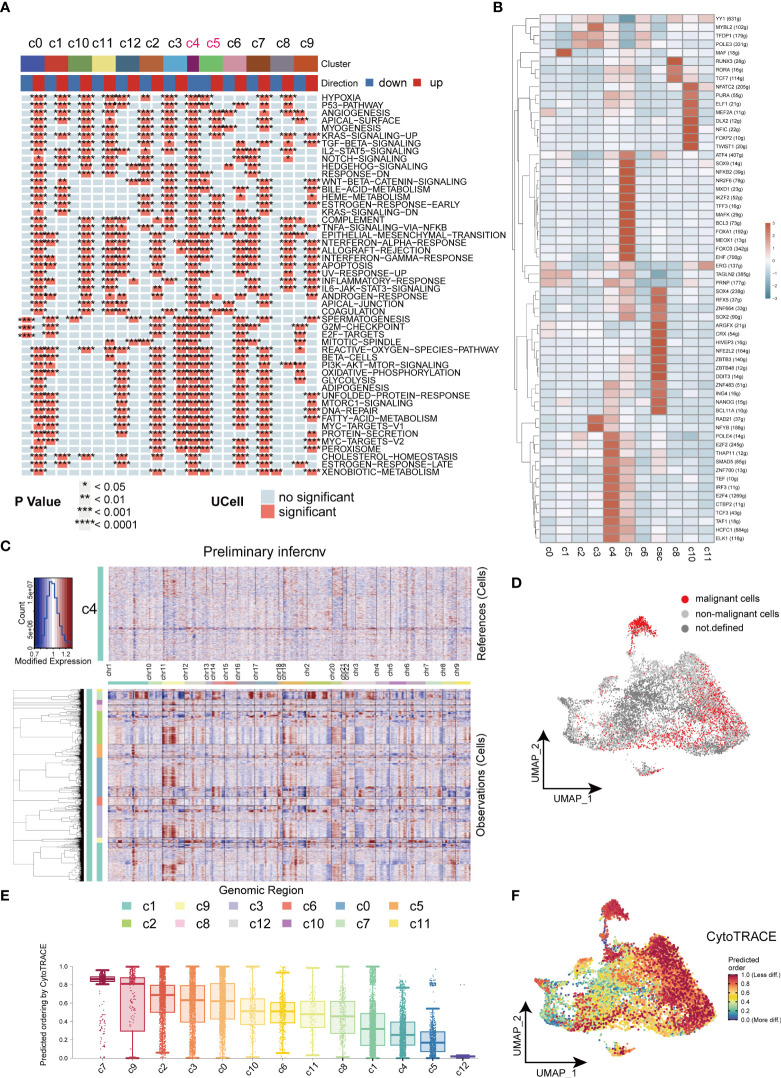
Heterogeneity in HNSCC. **(A)** Heatmap of cancer related signaling pathways enriched in different types of cell clusters (Hallmark gene set). **(B)** Heatmap of expressions of different transcription factors in cells of HNSCC. **(C)** Heatmap of the visualized inferCNV analysis. **(D)** UMAP plot of distribution of non-malignant cells in cancer cells. **(E)** Box plots of stemness among all the clusters in cancer cells. **(F)** UMAP plots of intensity of stemness in cancer cells. *P < 0.05, **P < 0.01, ***P < 0.001, ****P < 0.0001.

We further investigated the biological functions of different clusters by the irGSEA-UCell algorithm. Downregulation of tumor-associated signaling pathways were observed in C4 and C5 clusters and indicated they were non-malignant cell clusters (**Figure 4A**). In our results, CSCs highly expressed tumor stemness marker genes, SOX2 and NANOG, ZNF and ZBTB families. It has been reported ZNF and ZBTB families were extensively involved in cancer development and cell differentiation (33, 34). The substantial activation of ZNF and ZBTB families indicated their potential role in resulting in heterogeneity of tumors (**Figure 4B**). C4 cell clusters were described as non-malignant epithelial cells because of the downregulated cancer-related pathways, low levels of signaling communication and negative expression of CSCs markers. In this study, C4 was used as control to computational infer CNV status of cells in different clusters by inferCNV analysis. Similar CNV scores was obtained in C5, indicating cells of C5 are benign tumor cell populations. Strikingly, in inferCNV analysis, CSCs were characterized by its unique CNV amplification/deletion in different chromosomes compared with other tumor cells (**Figure 4C**). CopyKat analysis was performed to reveal the degree of malignancy of the different subpopulations of tumor cells. Our results indicated cells of C0, C1, C3 and C7 were densely enriched with malignant cells, and C7 was the densest region (**Figure 4D**). CytoTRACE was employed to estimate the differentiation potential within tumor cells and the results were mapped to the UMAP plot (**Figures 4E, F**). Cells of C7 and C9 of subsets exhibited strongest differentiation capacity, hinting that CSCs may be a determinant of intra-tumor heterogeneity (35).

### Functional diversity of CSCs at different differentiation stages

**Figure 5:**
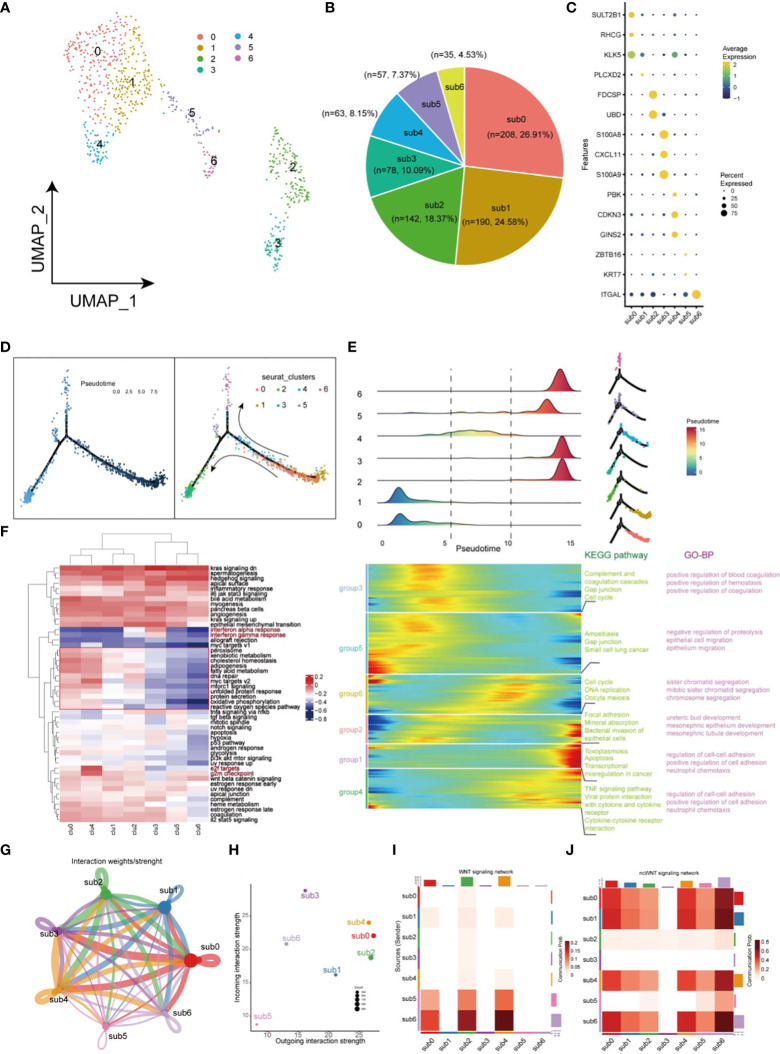
Landscape of characteristics of CSCs characterized by single-cell transcriptomic sequencing. **(A)** UMAP plot of 773 cells in CSCs. **(B)** Pie chart of the proportion of 7 types of cells in CSCs. **(C)** Bubble plot of the highest expressed genes within 7 types of cells in CSCs. The size of the dot represents the percentage of gene expression in the cell. **(D)** Pseudotime ordering of CSCs. The graph on the left is labeled with developmental time, while the graph on the right is labeled with cell state. **(E)** Plot of clustering of DEGs identified by the pseudo-temporal progression in CSCs. **(F)** Heatmap of 50 cancer-related pathways in 7 CSCs subsets using GSVA. **(G)** Weights/Strength of cell-cell interaction within 7 CSCs subsets. **(H)** Strength of output/input interaction in different CSCs subsets. **(I)** Plot of cells which interacted *via* WNT signaling pathways in CSCs. **(J)** Plot of cells which interacted *via* ncWNT signaling pathways in CSCs.

The uneven distribution of stemness markers, malignant markers, and CSCs markers in C7, C9, and C12 subpopulations indicated the heterogeneity of CSCs. Next, we focused on heterogeneity of CSCs. Seven cell clusters (subgroup0 - subgroup6, respectively) were generated based on the differential expression patterns of CSCs (**Figure 5A**). Pie charts and dot plots were employed to visualize the cell proportions and marker genes for each subpopulation of CSCs (**Figures 5B, C**). Then, pseudotime trajectory analysis were performed to sort each subgroup of CSCs along trajectories according to their expression and transition profiles. Subgroup0, 1 and 4 cells were at the beginning of the motor trajectory and could differentiation to Subgroup2/3 cells or Subgroup5/6 cells (**Figure 5D**). With the development of temporal trajectory, these sets of pseudotime-related genes at different developmental stages were divided into six groups involved in different KEGG pathways and GO biological processes (**Figure 5E**). Differentially expressed genes from different subsets were enriched in different signaling pathways which varied with the type of CSCs, showing significant phenotypic diversity (**Figure 5F**). Subgroup2/3 showed upregulation of inflammation-related pathways (Interferon-alpha and gamma response). In terms of interaction between different subgroup of CSCs, we found that sub0 and sub3 were the most active communicators in CSCs and send/receive the most signals, while subgroup5 was the least active communicator (**Figures 5G, H**). Especially, subgroup5 and 6 affected subgroups 0, 1, and 4 via WNT signaling pathway, while sub3 was not involved in cellular communication of ncWNT signaling network (**Figures 5I, J**).

### Naive CSCs are associated with a worse prognosis and clinicopathological progression in HNSCC patients

**Figure 6:**
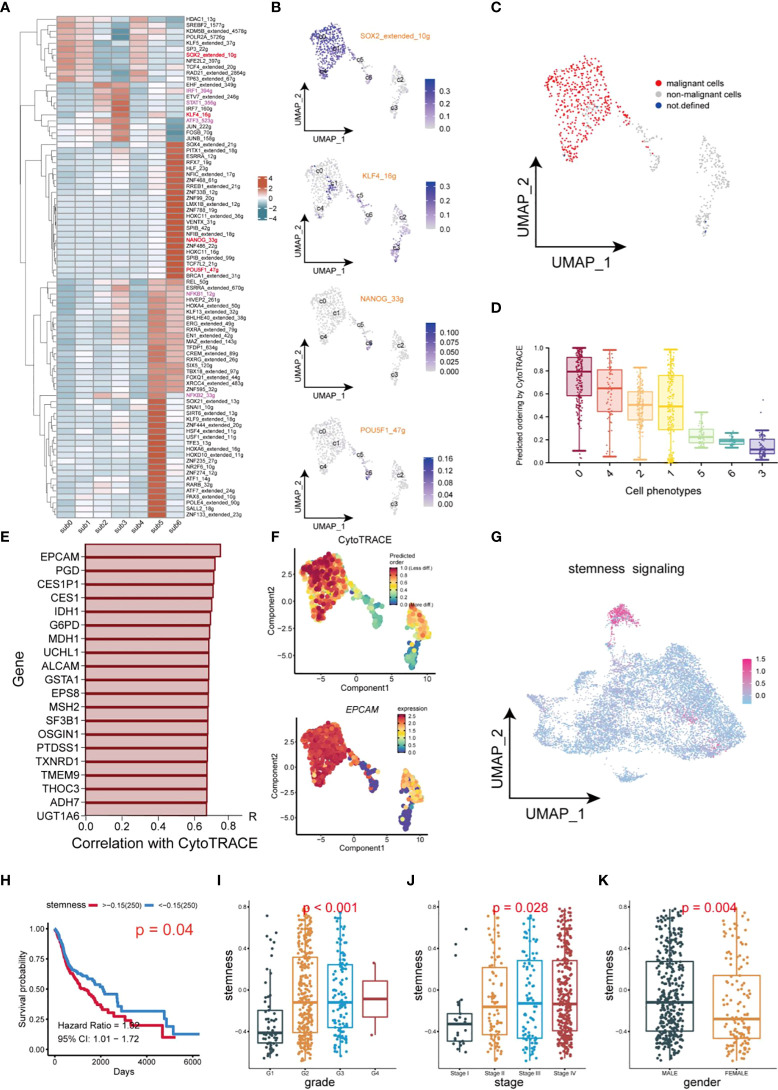
Transcription factor identification and stemness assessment in CSCs. **(A)** Heatmap of expression of transcription factors in different subsets of CSCs. **(B)** UMAP plot of distribution of SOX2, KLF4, NANOG, OCT4 (POU5F) in different subsets of CSCs. **(C)** UMAP plot of distribution of malignant cells and non-malignant cells in CSCs. **(D)** Box plots of stemness. **(E)** Top 20 genes which are positive correlation with CytoTRACE. **(F)** UMAP plot of intensity of stemness in different subsets of CSCs. **(G)** UMAP plot of distribution of stemness signals in cancer cells. **(H)** Plot of Kaplan-Meier survival analysis. **(I–K)** Box plot of analysis of the differences in stemness levels between different clinical parameters.

In this study, transcription factor (TF) networks of CSCs varied with the heterogeneity of subgroups of CSCs (**Figure 6A**). The activity of regulons was scored with AUCell. SOX2, KLF4, NANOG, and OCT4 are notorious for their specific expression in CSCs of HNSCC, which promoted stemness and tumor progression and lead to poor prognosis (12, 36, 37). Notably, these regulons differed considerably in transcriptional activity, because of the heterogeneity of CSCs (**Figure 6B**). Activation of Regulons (IRF1_394g, IRF7_160g, NFKB1_12g, NFKB2_33g and STAT1_356g) in subgroups 2 and 3 demonstrated a strong pro-inflammatory feature. The expression profile of CSCs using the copykat algorithm was employed to infer the genomic copy number distribution of individual cells thereby identifying naïve CSCs (subgroup 0,1 and 4) as the most malignant cell population (**Figure 6C**). Interestingly, regulon SOX2 and KLF4 are enriched in malignant/non-malignant respectively, suggesting that they may be a potential indicator for the identification of malignant cells within CSCs. The CytoTRACE scores of different populations within CSCs revealed the diversity of their differentiation capability (**Figure 6D**). Naïve CSCs had strongest differentiation potential, while subgroup5 and 6 cell populations showed lower. Combined application of CYTOTRACE and trajectory analysis revealed that the divergence between subgroup 2 and subgroup 3 ultimately exhibited an inflammation-related phenotype. Among the top 20 most positively correlated features of CytoTRACE, EPCAM was the most positively related factor (**Figure 6E**). The CytoTRACE score was mapped to the feature plot, and we observed an apparent enrichment of EPCAM with a trend of stemness consistent in naïve CSCs (**Figure 6F**). We used the top 20 features which most associated with CytoTRACE as the set of tumor stemness signature genes in the TCGA cohort and calculated the tumor stemness score for each sample by GSVA. The UMAP plot also revealed enrichment of tumor stemness signals, mainly in naïve CSCs (**Figure 6G**). So here, the stemness phenotype clearly reflects the presence of authentic CSCs in the tumor tissue. Higher tumor stemness scores were significantly associated with poorer overall survival, Grade grading, and Stage staging and were higher in men (**Figures 6H–K**), suggesting that the abundance of CSCs in HNSCC patients has meaningful effect on clinicopathological stages/grades and prognosis. Meanwhile, an external dataset was deployed to validate the survival analysis, which was consistent with our outcome (**Supplementary Figure 1C**).

## Discussion

CSCs are stem cell-like cells with self-renewal and multidirectional differentiation potential embedded in tumor tissue (35). CSCs are commonly accompanied with a high degree of immunosuppression, tumor recurrence, and metastases, resulting in becoming refractory tumors (38, 39). Therefore, eliminating CSCs is expected to be a new therapeutic target for the treatment of hyper-resistant cancers. So far, the understanding of the specific mechanism of CSCs in HNSCC still remains limited. Here, we used scRNA-seq to integrate multiple HNSCC patient data to generate single-cell transcriptome profiles and identified cancer stem cells. Our provide insights into the typing, biological characteristics, and regulatory signaling networks of CSCs in HNSCC.

In this study, we distinguished cancer cells from HNSCC tissues and isolated 13 subgroups. To better identify CSCs of HNSCC, a violin plot was employed to visualize the results of some markers’ expression of CSCs across different cell clusters (**Figure 2B**). The varied enrichment of OCT4 (POU5F1), SOX2, and NANOG in different types of cancer cells demonstrated the diversity of CSCs localization. C7, C9, and C12 exhibited similar incoming/outgoing cellular signaling in HNSCC tumor cells and expressed specific markers of CSCs, further confirmed the presence of CSCs. In general, CD44 is regarded as a non-negligible marker to identify CSCs in several cancer types, such as breast cancer and colorectal cancer (40, 41). However, OCT4, SOX2, and NANOG are obviously more suitable for the identification of CSCs in HNSCC (42). In our results, immune checkpoint molecules CD276, CD47 and LILRB2 were significantly up-regulated in CSCs. Previous studies have reported the upregulation of CD276 in tumor plays essential roles in tumorigenesis (11). Likewise, the highly abundance of CD47 enriched in CSCs enables CSCs to reduce NK cell- and myeloid-mediated cytotoxic activity ultimately and results in immune escaping (43, 44). The HLA-G-LILRB2 axis also has been identified to participate in the communication between CSCs and myeloid cells (**Figure 3F**), suggesting that CSCs may be involve in immune escape-related signaling via promoting the maturation and differentiation of different types of myeloid cells (45). In conclusion, CSCs successfully evade elimination from immune cells by up-regulating some immune checkpoints to form an immunosuppressive environment.

Unexpectedly, the C8 population of cancer cells expressed T-cell markers (CD3D, CD3E, CD7), which is recognized as a dual identity of T cells and tumor cells involved in the role of the TME. For instance, MIF signal is the strongest signal for CSCs to communicate with other cells in TME, and C8 displayed characteristics of immune cells and cancer cells in tumor by expressing different receptors (**Figure 3A**). Previous studies have found that T cell subsets are highly enriched for malignant epithelial markers leading perturbation of T cell function via cell-cell signaling (46). In this study, the origination of C8 subgroup and whether it affects T cell and tumor cell function remain to be investigated.

The inferred CNAs analysis revealed great genomic alterations in CSCs in HNSCC, unlike other tumor cells, including gain of chromosomes 2, 3, 12 and loss of chromosome 11(**Figure 4C**). Meantime, a large amount of malignant cells are computationally inferred to be present in CSCs, illustrating the extremely genomic instability of CSCs (**Figure 4D**). CSCs were divided into 7 subgroups to further unveil the heterogeneity within CSCs. Intriguingly, inflammation-related factors S100A8, S100A9 are highly expressed in subgroup3 of CSCs. Further GSVA analysis suggested the signaling pathways varies with the type of CSCs in tumors, showing significant functional diversity of CSCs (**Figure 5F**). For example, subgroup 2 and 3 both exhibited inflammation-related signals (Interferon alpha/gamma response). In the crosstalk of IRF, NFKB and JAK/STAT pathways, HBV invasion in HNSCC is highly likely to be a decisive factor in the induction of naive CSCs into inflammation-associated CSCs. Low stemness CSCs (subgroups 5 and 6) could promote the proliferation or differentiation high stemness CSCs (subgroups 0, 2 and 4) through WNT signaling pathway, elucidating a monumental way for CSCs to maintain high stemness (29). In addition, CSCs also participate in cell directed migration of CD44 cells, endothelial cells, and fibroblasts *via* ncWNT signaling pathway, ultimately leading to the formation of the metastatic microenvironment (29).

## Conclusion

In this study, we delineated a comprehensive single-cell transcriptomic atlas of CSCs in the TME of HNSCC for the first time. Our results revealed the heterogeneity of CSCs in HNSCC and elucidated the important roles of CSCs in the formation of immunosuppressive TME. This study also investigated the functions of different types of CSCs and the complex regulatory networks between CSCs and other tumor cells. Our study about heterogeneity of CSCs in HNSCC can bring new insights for the treatment of HNSCC.

## Data availability statement

The original contributions presented in the study are included in the article/**Supplementary Material**. Further inquiries can be directed to the corresponding authors.

## Author contributions

All authors listed have made a substantial, direct and intellectual contribution to the work, and approved it for publication.

## Funding

This work was supported by National Natural Science Foundation of China (No. 81972643, No. 82172962), Sichuan Science and Technology Project (2021YJ0201), Luxian People’s Government and Southwest Medical University Scientific and Technological Achievements Transfer and Transformation Strategic Cooperation Project (2019LXXNYKD-07) and Science and Technology Program of Luzhou, China (No. 21CGZHPT0001).

## Conflict of interest

The authors declare that the research was conducted in the absence of any commercial or financial relationships that could be construed as a potential conflict of interest.

## Publisher’s note

All claims expressed in this article are solely those of the authors and do not necessarily represent those of their affiliated organizations, or those of the publisher, the editors and the reviewers. Any product that may be evaluated in this article, or claim that may be made by its manufacturer, is not guaranteed or endorsed by the publisher.
