# Supplementary material for: Complex interaction and heterogeneity among cancer stem cells in head and neck squamous cell carcinoma revealed by single-cell sequencing
Source: Front Immunol. 2022 Nov 14;13:1050951. doi: 10.3389/fimmu.2022.1050951 (PMC9701714; doi:10.3389/fimmu.2022.1050951)
Supplement: Supplementary file 1 [file DataSheet_1.docx]

Supplementary Material

## Supplementary Figures


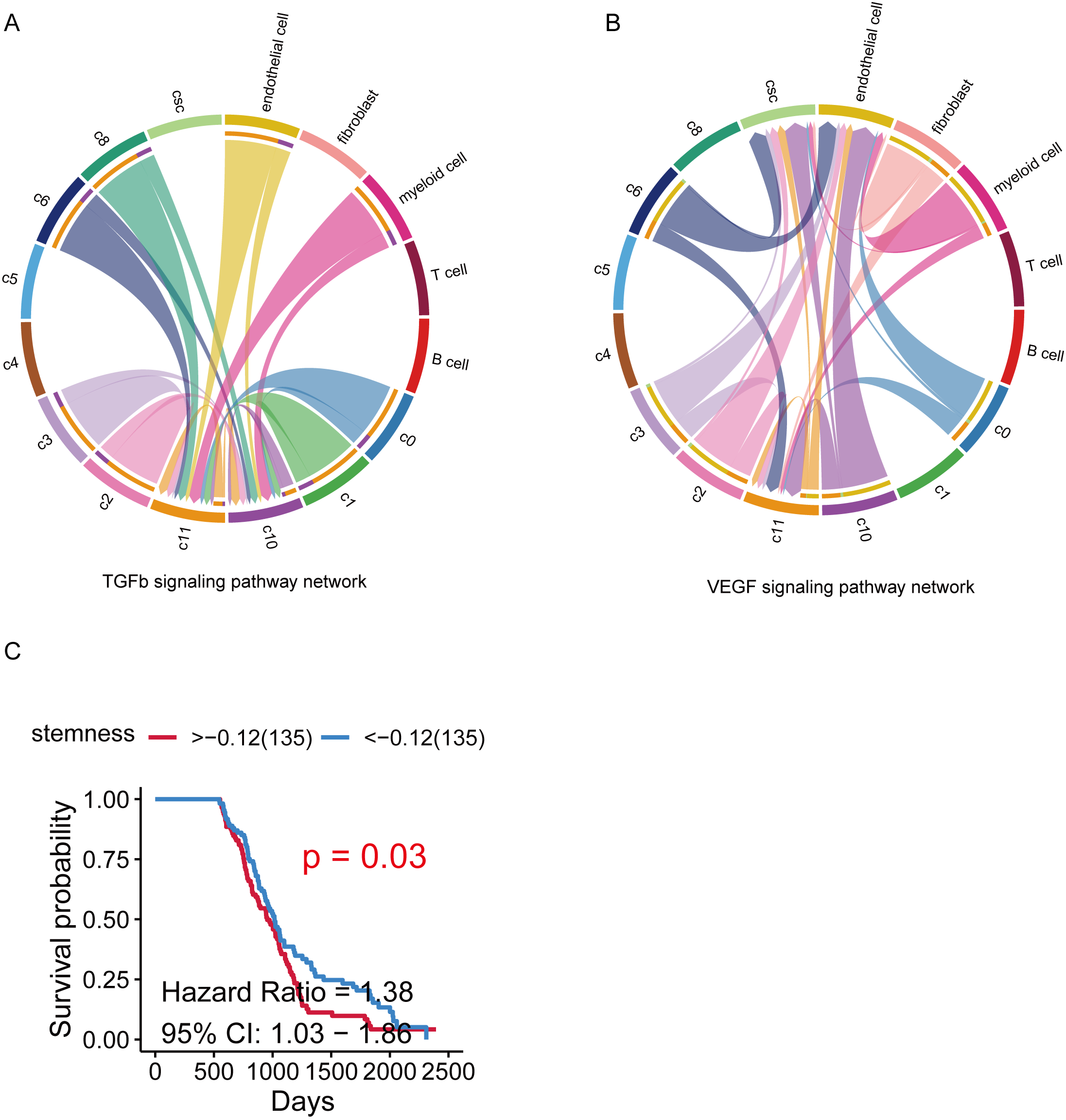


**Supplementary Figure 1.** **Validation of survival analysis and** **Cellchat analysis of TGF-β and VEGF signaling networks in TME within HNSCC.** (A) Plot of cells which interacted via TGF-β signaling pathways in HNSCC. (B) Plot of cells which interacted via VEGF signaling pathways in HNSCC. (C) In the GSE65858 cohort, Kaplan-Meier analysis of prognosis in HNSCC patients with High/Low stemness.
